# Морфологические характеристики аденом гипофиза в рамках фенокопий синдрома множественных эндокринных неоплазий 1 типа

**DOI:** 10.14341/probl12815

**Published:** 2021-12-30

**Authors:** Д. А. Трухина, Е. О. Мамедова, А. М. Лапшина, Е. В. Васильев, А. Н. Тюльпаков, Ж. Е. Белая

**Affiliations:** Национальный медицинский исследовательский центр эндокринологии; Национальный медицинский исследовательский центр эндокринологии; Национальный медицинский исследовательский центр эндокринологии; Национальный медицинский исследовательский центр эндокринологии; Национальный медицинский исследовательский центр эндокринологии; Медико-генетический научный центр имени академика Н.П. Бочкова; Национальный медицинский исследовательский центр эндокринологии

**Keywords:** менин, синдром множественных эндокринных неоплазий 1 типа, фенокопия, аденома гипофиза, гистология

## Abstract

**ОБОСНОВАНИЕ:**

ОБОСНОВАНИЕ. Синдром множественных эндокринных неоплазий 1 типа (МЭН  1) — это редкое аутосомно-доминантное заболевание, обусловленное мутациями в гене MEN1, кодирующем белок менин. В случае выявления у пациента фенотипа МЭН 1 при отсутствии мутаций в гене MEN1 состояние расценивается как фенокопия данного синдрома. Хотя в понимании функции менина был достигнут значительный прогресс, его значение в онкогенезе эндокринных желез все еще выясняется. Благодаря ключевой роли этого гена в физиологических и патологических процессах оценка экспрессии менина может дать ценную информацию.

**ЦЕЛЬ:**

ЦЕЛЬ. Определить, имеются ли какие-либо различия в экспрессии менина в тканях аденом гипофиза (АГ) у пациентов с фенокопиями синдрома МЭН 1 и генетически подтвержденным МЭН 1 по сравнению с их спорадическими формами.

**МАТЕРИАЛЫ И МЕТОДЫ:**

МАТЕРИАЛЫ И МЕТОДЫ. Проведено одномоментное одноцентровое исследование: иммуногистохимическая (ИГХ) оценка экспрессии менина и типа секреции опухолей гипофиза пациентов с генетически подтвержденным МЭН 1 (гМЭН 1), фенокопиями МЭН 1 (фМЭН 1) и спорадической акромегалией (СА), оперированных в 2008–2020 гг. ИГХисследование выполнено при использовании антител к менину, пролактину, соматотропному, адренокортикотропному, фолликулостимулирующему, тиреотропному гормонам, Pit-1, T-box, ERA на заранее подготовленных срезах толщиной 3–4 мкм.

**РЕЗУЛЬТАТЫ:**

РЕЗУЛЬТАТЫ. В исследование было включено 35 образцов опухолей гипофиза: гМЭН 1 — 9 образцов, фМЭН 1 — 12, CA — 14. Пациенты трех групп были сопоставимы по полу, размерам АГ, приему лекарственных препаратов. Группа гМЭН 1 отличалась от фМЭН 1 и СА по возрасту (р=0,0005). У пациентов с гМЭН 1 экспрессия менина варьировала от отсутствия окрашивания (5/9) до интенсивного окрашивания цитоплазмы. В группе фМЭН  1 в основном присутствовала цитоплазматическая экспрессия менина (11/12). В группе СА окраска отсутствовала в 1 случае, ядерная экспрессия была выявлена в 6/14 случаев. Группа фМЭН  1 показала значительно большую цитоплазматическую экспрессию менина, чем в группе гМЭН  1 (p=0,006). Группа гМЭН 1 также отличалась от группы СА по экспрессии менина (р=0,012). Статистически значимых различий между группами фМЭН 1 и СА выявлено не было (p=0,049).

**ЗАКЛЮЧЕНИЕ:**

ЗАКЛЮЧЕНИЕ. По результатам исследования оценки экспрессии менина выявлено, что экспрессия в целом сохранена в АГ у фМЭН 1 и СА, хотя и с разной локализацией в структуре клетки (ядро и/или цитоплазма). В то же время экспрессия менина сильно варьирует в АГ у пациентов с гМЭН 1. По полученным данным можно предположить, что патогенез АГ при фМЭН 1 и СА может иметь сходство, однако, по всей видимости, имеются факторы, способствующие появлению нескольких опухолей эндокринных желез у одного человека с фМЭН 1. Для понимания процесса необходимо дальнейшее исследование ассоциированных с МЭН 1 генов, эпигенетических факторов, сигнальных путей, в которых участвует менин.

## Abstract (en)

**BACKGROUND:**

BACKGROUND: Multiple endocrine neoplasia type 1 (MEN 1) is a rare autosomal dominant disorder caused by mutations in the MEN1 gene, which encodes the menin protein. If a patient has the MEN 1 phenotype in the absence of mutations in the MEN1 gene, the condition is classified as a phenocopy of this syndrome. Although significant progress has been made in understanding the function of menin, its role in the oncogenesis of the endocrine glands is still being elucidated. Due to its key role in physiological and pathological processes, the assessment of the menin expression can provide valuable information.

**AIM:**

AIM: to determine whether there are any differences in the expression of menin in the pituitary adenomas (PA) in patients with phenocopy of MEN 1 (phMEN 1) and genetically confirmed MEN 1 (gMEN 1) compared with their sporadic forms.

**MATERIALS AND METHODS:**

MATERIALS AND METHODS: immunohistochemical assessment of the menin expression was carried out in PA of patients with gMEN 1, phMEN 1 and sporadic acromegaly (SA), surgically treated in 2008–2020. IHC was performed using antibodies to menin, PRL, GH, ACTH, FSH, TSH, Pit-1, T-box, ERA on previously prepared histological section.

**RESULTS:**

RESULTS: The study included 35 samples of PA: gMEN 1 — 9 samples, phMEN 1 — 12 (somatotropinomas + PHPT); CA — 14  samples. The patients were comparable by gender, adenoma size, and drug intake. The gMEN  1 group differed from phMEN 1 and SA by age (p = 0.0005). In patients with gMEN 1, the expression of menin varied from no staining (5/9) to intense cytoplasm staining. Cytoplasmic expression of menin was mainly present (11/12) in the phMEN 1. In the SA group, there was no staining in 1 case; nuclear expression was detected in 6/14 cases. The phMEN  1 group showed significantly higher cytoplasmic expression of menin than the gMEN  1 group (p = 0.006). The gMEN 1 group also differed from the SA group (p = 0.012). There were no statistically significant differences between the phMEN 1 and SA groups (p = 0.049).

**CONCLUSION:**

CONCLUSION: It was revealed that the menin expression, in general, is retained in phMEN 1 and SA groups, although with different localization in the cell structure (nucleus and / or cytoplasm). At the same time, the expression of menin varies greatly in patients with gMEN 1. According to the data obtained, it can be assumed that the pathogenesis of PA in phMEN 1 and SA may have similarities; however, there could be factors contributing to the appearance of several tumors of the endocrine glands in one person with phMEN 1. To understand this process, it is necessary to further study the genes associated with MEN 1, epigenetic factors, signaling pathways in which menin is involved.

## ВВЕДЕНИЕ

Аденомы гипофиза (АГ) — это доброкачественные опухоли передней доли гипофиза, составляющие до 10% всех внутричерепных опухолей и встречающиеся в 15–20% случаев по данным аутопсии или в качестве рентгенологических находок [1]. АГ бывают как спорадическими (95%), так и в рамках наследственных синдромов (5%), таких как семейные изолированные аденомы гипофиза (FIPA), синдром множественных эндокринных неоплазий 1 типа (МЭН 1), Х-сцепленный акрогигантизм, синдром 3P и др. [2][3].

Синдром МЭН 1 — это редкое аутосомно-доминантное заболевание, обусловленное мутациями в гене MEN1, кодирующем белок менин. У пациентов с синдромом МЭН 1 АГ встречаются примерно в 40% всех случаев [4]. Большинство АГ при синдроме МЭН 1 представлены пролактин-секретирующими (42–62%) или гормонально-неактивными опухолями (15–42%); АГ, секретирующие гормон роста и адренокортикотропный гормон (АКТГ), встречаются реже (6,5–9 и 3–4% случаев соответственно) [5]. АГ также имеют более высокую вероятность сочетанной секреции нескольких гормонов, считаются более агрессивными и более резистентными к проводимому лечению. Соматические мутации гена MEN1 редко можно обнаружить в спорадических опухолях гипофиза [6].

В 10–30% семейных случаев МЭН 1 и 60–80% спорадических случаев синдрома мутации в гене MEN1 не выявляются, что может быть связано с крупными делециями данного гена, мутациями в промоторе или других нетранслируемых областях, которые обычно не анализируются в «рутинных» генетических исследованиях [7]. В случае выявления у пациента фенотипа МЭН 1 при отсутствии мутаций в гене MEN1 состояние расценивается как фенокопия данного синдрома. Наиболее часто при фенокопиях МЭН 1 имеется сочетание АГ (чаще — соматотропиномы) и опухоли околощитовидной железы (приводящей к развитию первичного гиперпаратиреоза (ПГПТ)). Причина сочетания нескольких эндокринных МЭН 1-ассоциированных опухолей у таких пациентов остается неизвестной. Как возможные причины могут быть рассмотрены мутации в других, еще не установленных генах, эпигенетические изменения, а также случайное сочетание нескольких опухолей у одного пациента [8][9].

Ген MEN1 локализован на длинном плече хромосомы 11q13 и кодирует многофункциональный белок менин [10]. Менин выполняет интегральные ядерные функции, поскольку он напрямую взаимодействует с ДНК (независимым от последовательности образом) и с белками, ответственными за транскрипцию (активацию или супрессию), передачу сигналов клетки, регуляцию репарации ДНК и структурную целостность клетки [11]. В качестве опухолевого супрессора менин, как было выявлено, связывает транскрипционный фактор АР-1 JunD и взаимодействует со SMAD3, каноническим эффектором пути TGF-β, ингибируя рост клеток [12][11]. Важная роль менина в ядре подчеркивается в исследованиях на мышах in vivo, которые показали, что менин регулирует рост клеток, уменьшая экспрессию ингибиторов циклин-зависимой киназы p18Ink4c и p27Kip1, чтобы снизить активность циклин-зависимой киназы 2 и ограничить пролиферацию клеток [13][14].

Хотя в понимании функции менина был достигнут значительный прогресс, его роль в онкогенезе эндокринных желез все еще выясняется. Благодаря своей ключевой роли в физиологических и патологических процессах оценка менина может дать ценную информацию в опухолеобразовании эндокринных желез, в том числе и аденом гипофиза. Были проведены работы, в которых оценивалась экспрессия менина в АГ. В 2002 г. С. Wrocklage и соавт. исследовали экспрессию менина в 11 спорадических опухолях гипофиза и 4 нормальных гипофизах с помощью антител к менину и выявили большую экспрессию менина в опухолевых тканях, чем в нормальных тканях [15]. М. Theodoropoulou и соавт. проанализировали экспрессию менина в 7 нормальных гипофизах и 68 спорадических АГ. В гипофизах без опухоли была обнаружена выраженная ядерная экспрессия менина; в спорадических АГ экспрессия менина была различной, при этом высокий процент случаев демонстрировал значительное снижение экспрессии менина по сравнению с нормальным гипофизом; в случае пролактин-продуцирующей карциномы экспрессия менина отсутствовала [16]. К. Kooblall и соавт. исследовали экспрессию менина в семье с мутацией в гене MEN1 в нетранслируемой области 5’UTR. При анализе белковых лизатов, полученных из трансформированных вирусом Эпштейна–Барр лимфобластоидных клеток, выделенных от пациентов с МЭН 1 и относительно здоровых индивидуумов, ученые выявили снижение экспрессии менина у всех в семье с мутацией по сравнению с двумя здоровыми родственниками и двумя неродственными нормальными контролями [17].

Учитывая отсутствие исследований в группах фенокопий синдрома МЭН 1, в данной работе мы решили выяснить, существуют ли какие-либо различия в экспрессии менина в тканях АГ у пациентов с фенокопиями синдрома МЭН-1 (фМЭН 1) и генетически подтвержденным МЭН 1 (гМЭН 1) по сравнению с их спорадическими формами. Знание того, является ли экспрессия менина одинаковой или отличной между группами, может дать представление о роли менина в онкогенезе АГ.

## МАТЕРИАЛЫ И МЕТОДЫ

Дизайн исследования

Проведено наблюдательное одномоментное одноцентровое исследование.

Место и время проведения исследования

Исследование проводилось в ФГБУ «НМИЦ эндокринологии» Минздрава России. Были использованы образцы тканей гипофиза пациентов, оперированных с 2008 по 2020 гг.

Критерии соответствия

Критерии включения: образцы тканей АГ, полученных в ходе трансназальной транссфеноидальной аденомэктомии пациентов с гМЭН 1, фМЭН 1, со спорадической акромегалией (СА).

Критерии исключения: злокачественные новообразования, лучевая терапия, отсутствие генетического тестирования у пациентов с фенотипом МЭН 1.

Способ формирования выборки: выборки были сформированы путем сплошного включения наблюдений. Предварительного расчета выборки не проводилось. Пациенты были разделены на 3 группы: набор в первые две группы определялся результатом генетического исследования, 3-я группа представляла собой СА, куда были включены пациенты с соматотропиномами и отсутствием других эндокринных или неэндокринных образований.

Генетическое тестирование

Генетическое тестирование было проведено всем пациентам с фенотипом синдрома МЭН. Секвенирование было проведено по Сэнгеру и методом высокопроизводительного параллельного секвенирования (next-generation sequencing — NGS) панели генов-кандидатов (MEN1, CDKN1A, CDKN1B, CDKN1C, CDKN2A, CDKN2C, CDKN2D, AIP, SDHA, SDHB, SDHC, SDHD, PRKAR1A, GNAS, PRKCA, POU1F1, CASR, CDC73). При отсутствии мутации в гене MEN1 проведен MLPA (метод мультиплексной амплификации лигированных зондов) для обнаружения крупных делеций/дупликаций кодирующей области MEN1. Геномную ДНК выделяли из лейкоцитов периферический крови стандартным методом (набор Pure Link, Genomic DNA Mini Kit, Life Technologies, США). Подготовка библиотек проводилась в соответствии с рекомендациями производителей. Секвенирование осуществлялось на Illumina MiSeq (Illumina, США). Биоинформатическая обработка результатов секвенирования проводилась с помощью программных модулей Genome Analysis ToolKit (GATK) ver. 4.1.2.0 (Broad Institute, Cambridge, MA, USA). Для аннотирования вариантов нуклеотидной последовательности использовалась программа ANNOVAR (http://annovar.openbioinformatics.org). Оценка патогенности вариантов нуклеотидной последовательности проводилась согласно международным и российским рекомендациям [18][19].

В группу фМЭН 1 включались пациенты при отсутствии выявления мутации во всех вышеперечисленных генах. В группе СА генетическое тестирование не проводилось ввиду отсутствия клинических показаний для проведения исследования.

Иммуногистохимическое исследование

С парафиновых блоков образцов тканей опухолей гипофиза, полученных в ходе трансназальной транссфеноидальной аденомэктомии, были изготовлены срезы толщиной 3–4 мкм, которые наносили на адгезивные стекла (Menzel GmbH&Co KG, Германия). Депарафинирование, демаскировка антигенов проводились при помощи высоко- и низко рН-буферов (Leica). Антитела (АТ), используемые для определения типа секреции АГ и экспрессии менина: АТ к менину (Abcam ab2605), которые связывались с частью С-конца менина человека; АТ к пролактину, соматотропному, адренокортикотропному, фолликулостимулирующему, тиреотропному гормонам (Dako), Pit-1, T-box (Novus Bio), ERA (Abcam).

При оценке экспрессии менина использовалась 4-балльная система оценки: отсутствие окраски обозначалось как 0; цитоплазматическая окраска в зависимости от степени выраженности окрашивания обозначалась как ц1 (слабая), ц2 (средняя), ц3 (сильная); ядерная окраска в зависимости от степени выраженности окрашивания обозначалась как я1, я2, я3 соответственно.

Этическая экспертиза

Протокол исследования одобрен на заседании локального этического комитета ФГБУ «НМИЦ эндокринологии» Минздрава России от 10 марта 2021 г. (протокол №4).

Статистический анализ

Для статистической обработки материала использовались программы Statistica 13.3 (StatSoft США), IBM SPSS 23. Данные описательной статистики представлены в виде медианы, а также 25-го и 75-го перцентилей. Для описания качественных данных рассчитывали абсолютные (n) и относительные значения (%). Нормальность распределения проверялась критерием Шапиро–Уилка. Связь между количественными показателями устанавливали, используя непараметрический метод Краскела–Уоллиса ANOVA, с поправкой на множественные сравнения Бонферрони (р<0,017). Для анализа связей между категориальными переменными использовали критерий χ-квадрат Пирсона и точный критерий Фишера. Статистически значимыми считали различия при p<0,05.

## РЕЗУЛЬТАТЫ

Характеристика пациентов

В исследование было включено 35 образцов опухолей гипофиза. В группу гМЭН 1 вошли 9 образцов, в группу фМЭН 1 — 12; в группу CA — 14 образцов.

Пациенты трех групп были сопоставимы по полу, размерам АГ, приему лекарственных препаратов. Группа гМЭН 1 отличалась от фМЭН 1 и СА по возрасту (р=0,0005): пациенты в этой группе были моложе на момент проведения трансназальной транссфеноидальной аденомэктомии (табл. 1).

АГ в группе гМЭН 1 представлены 3 кортикотропиномами, 2 соматотропиномами, 1 молчащей гонадотропиномой, 1 пролактиномой; две опухоли секретировали несколько гормонов: АКТГ-ПРЛ и ТТГ-ПРЛ. ПГПТ диагностирован у 8 из 9 пациентов. Более подробно компоненты синдрома МЭН 1 в группе гМЭН 1 представлены в таблице 2.

В группе фМЭН 1 все АГ являлись соматотропиномами: 5 — редкогранулированных соматотропином (РГС), 7 — плотногранулированных соматотропином (ПГС). У всех пациентов группы фМЭН 1 был установлен диагноз ПГПТ (паратгормон 104,85 пг/мл [ 91,63; 165,85]; кальций, скорректированный по альбумину, 2,71 ммоль/л [ 2,61; 2,78]). У 3 пациентов, кроме поражения гипофиза и околощитовидных желез, были выявлены другие образования (табл. 3).

**Table:** Таблица 1. Клинические характеристики исследуемых групп

Показатель	гМЭН 1 (1)n=9	фМЭН 1 (2)n=12	СА (3)n=14	р-критерий
Возраст на момент проведения операции, лет	36 [ 27; 47]	59 [ 56; 65]	56 [ 53; 62]	0,0005р1–2= 0,0005р2–3=1,00р1–3= 0,0067
Пол, жен., n (%)	10 (71,4)	10 (83,3)	6 (66,6)	0,65
Размер аденомы, микро, %	4 (44,4)	3 (25)	6 (42,8)	0,56
Прием лекарственных препаратов:-аналоги соматостатина, n (%)-агонисты дофаминовых рецепторов, n (%)	–5 (55,6)	5 (41,7)–	4 (28,6)2 (14,3)	0,02

**Table:** Таблица 2. Характеристика пациентов в группе генетически подтвержденного синдрома множественных эндокринных неоплазий 1 типа

№	Возраст на момент проведения операции; пол	Образования	Гистологическая характеристика АГ
1	36; ж	Соматотропинома; гиперплазия левого надпочечника; ГН НЭО ПЖЖ; ПГПТ; образование правой молочной железы	ПГС
2	27; ж	Кортикотропинома; ПГПТ; ГН НЭО ПЖЖ	ПГК
3	47; ж	Кортикопролактинома; ПГПТ; гастринома; ГН НЭО ПЖЖ; объемное образование правого яичника	Двойная кортико- и пролактинома
4	20; м	Кортикотропинома	ПГК
5	57; м	Соматотропинома; ПГПТ	ПГС
6	37; ж	Кортикотропинома; ПГПТ; двусторонняя гиперплазия надпочечников	ПГК
7	33; ж	Пролактинома; ПГПТ	ПГП
8	13; м	Тиреопролактинома; ПГПТ	Полигормональная Рit1 позитивная опухоль гипофиза (ТТГ+ПРЛ)
9	49; ж	Гонадотропинома; ПГПТ; менингиомы правой лобно-теменной области	Молчащая гонадотропинома

**Table:** Таблица 3. Характеристика пациентов с множественными образованиями в группе фенокопий синдрома множественных эндокринных неоплазий 1 типа

№	Возраст на момент проведения операции; пол	Образования	Гистологическая характеристика АГ
1	59, ж	Соматотропинома; ПГПТ; ГНО надпочечника; ГН НЭО ПЖЖ, нейрофиброма правого забрюшинного пространства; гемангиомы лобной кости; ТhXI позвоночника	ПГС
2	68, ж	Соматотропинома; ПГПТ; ГНО обоих надпочечников; аденокарцинома правой почки	ПГС
3	72, ж	Соматотропинома; ПГПТ; кистозное образование головки ПЖЖ; кисты обеих почек, печени; гиперплазия обоих надпочечников	ПГС

В группе СА по данным гистологической характеристики АГ были разделены на 5 РГС и 9 ПГС. У пациентов не было обнаружено других эндокринных и неэндокринных заболеваний. Уровень кальция, скорректированного по альбумину, равнялся 2,41 ммоль/л [ 2,36; 2,45].

Экспрессия менина по данным ИГХ-исследования

Результаты окрашивания на менин и сравнение результатов оценки экспрессии менина между тремя группами представлены в таблице 4. В качестве положительного контроля в работу были взяты образцы ткани нормального гипофиза, полученные на аутопсии и после оперативного вмешательства без признаков патологии. Также использовалась ткань нормальной поджелудочной железы (ПЖЖ) после оперативного вмешательства по поводу нейроэндокринной опухоли (НЭО) ПЖЖ. В ткани гипофиза обнаружена цитоплазматическая экспрессия менина; в эндокринной части ткани ПЖЖ (островки Лангерганса) — ядерная экспрессия.

**Table:** Таблица 4. Результаты окрашивания на менин в группах фенокопиями, генетически подтвержденным синдромом множественных эндокринных неоплазий 1 типа и спорадической акромегалией, СА.

Окраска/Группы	гМЭН 1 (1)n=9	фМЭН 1 (2)n=12	СА (3)n=14	p-критерий
Ядерная	0	1 (с)	6 (с-3; ср-3)	0,001р1–2=0,006р1–3=0,012р2–3=0,049
Цитоплазматическая	4 (с-3; ср-1)	11 (с-1; ср-7; сл-3)	7 (ср)
Отсутствие	5	0	1

Группа фМЭН 1 показала значительно большую цитоплазматическую экспрессию менина (рис. 1), чем в группе гМЭН 1 (p=0,006). Группа гМЭН 1 также отличалась от группы СА по экспрессии менина: ядерной окраски в группе 1 не было выявлено ни в одном случае (рис. 2), обнаруженная цитоплазматическая экспрессия менина в 3 образцах была слабой степени выраженности, в одном — средней (p=0,012) (рис. 3).

Статистически значимых различий между группами фМЭН 1 и СА выявлено не было (p=0,049). Хотя между этими двумя группами не было обнаружено различий, интересно, что в группе фМЭН 1 только в одном случае была выявлена слабая ядерная экспрессия менина, тогда как в группе СА ядерная экспрессия была выявлена в 6 случаях (3 только в ядре, 3 и в ядре, и в цитоплазме) (рис. 4).

**Figure:**
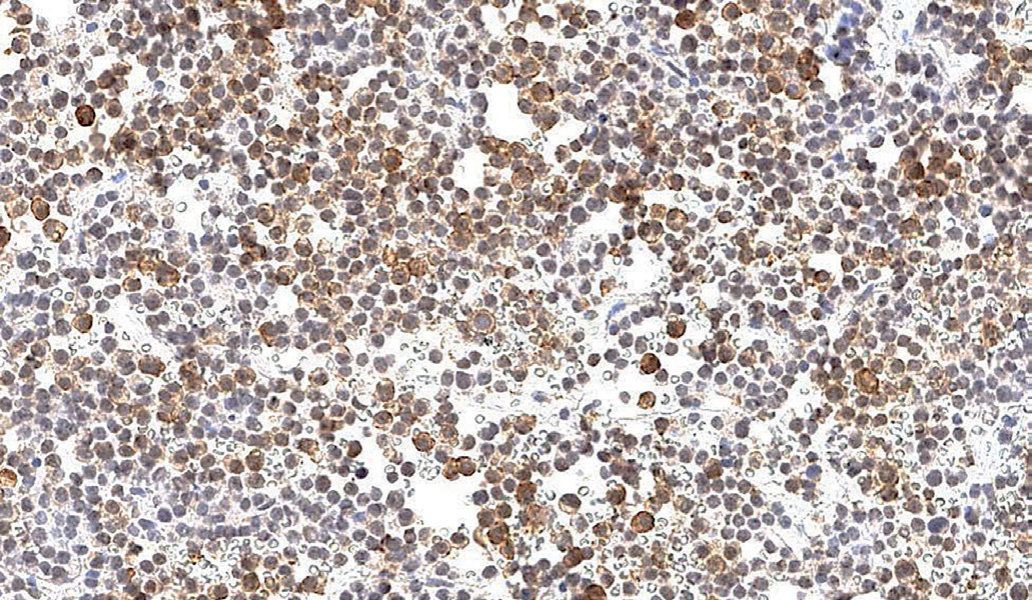
Рисунок 1. Цитоплазматическая экспрессия менина в образце ткани аденомы гипофиза у пациента с фенокопией синдрома множественных эндокринных неоплазий 1 типа, х200.

**Figure:**
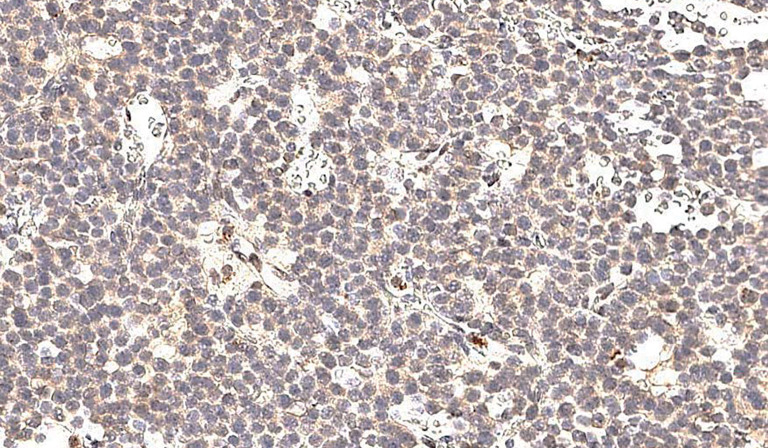
Рисунок 2. Отсутствие экспрессии менина и в ядре, и в цитоплазме в образце ткани аденомы гипофиза у пациента с генетически подтвержденным синдромом множественных эндокринных неоплазий 1 типа, х200.

**Figure:**
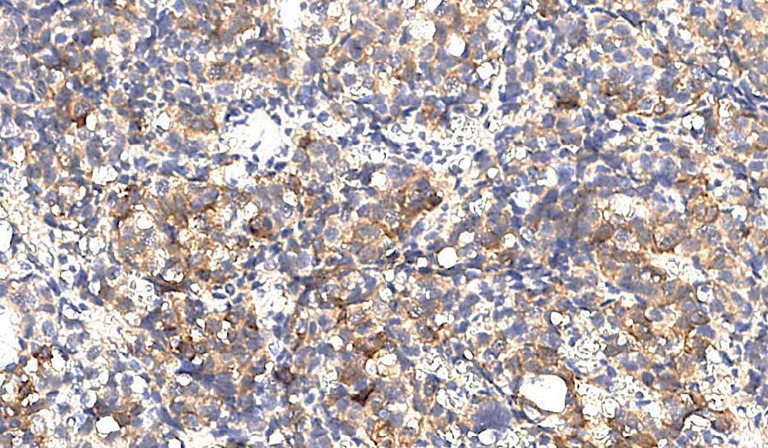
Рисунок 3. Слабая цитоплазматическая экспрессия менина в образце ткани аденомы гипофиза у пациента с генетически подтвержденным синдромом множественных эндокринных неоплазий 1 типа, х200.

**Figure:**
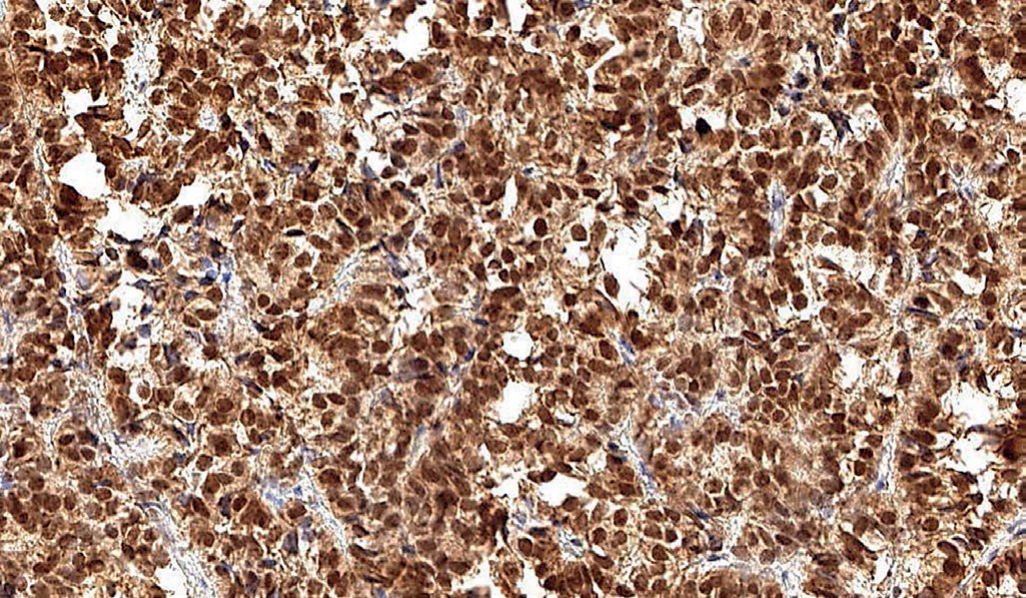
Рисунок 4. Ядерная и цитоплазматическая экспрессия менина в образце ткани аденомы гипофиза у пациента со спорадической акромегалией.

## ОБСУЖДЕНИЕ

В нашем исследовании по оценке экспрессии менина в тканях АГ в группах с гМЭН 1 и его фенокопиями по сравнению с группой СА в большинстве случаев экспрессия менина отсутствовала в группе с гМЭН 1 (5/9), однако мы также выявили отсутствие экспрессии в 1 случае в группе СА. Цитоплазматическая экспрессия менина, преимущественно слабой степени интенсивности, выявлена в 4 образцах в группе гМЭН 1 и в 7 образцах в группе фМЭН 1; по данным некоторых исследований, экспрессия менина может наблюдаться и при наличии мутаций в гене MEN1 [20][21]. A. Sonoda, и соавт. в своей работе исследовали клинико-патологические особенности и экспрессию менина в НЭО ПЖЖ. Ими были проанализированы 7 НЭО ПЖЖ, ассоциированных с мутацией в гене MEN1, и 70 спорадических НЭО ПЖЖ. В 6/7 НЭО ПЖЖ с МЭН 1 отсутствовала ядерная экспрессия менина, в 2 — была выявлена цитоплазматическая экспрессия; в 21 спорадических НЭО ПЖЖ также отсутствовала ядерная экспрессия менина, что, по мнению авторов, могло быть, вероятно, связано с включением образцов с мутациями в гене MEN1 [20]. Вероятнее всего, в нашей работе отсутствие экспрессии в одном случае группы СА может быть также ввиду возможной соматической мутации в MEN1 или эпигенетических изменений.

V. Corbo и соавт. в своем исследовании изучали генетические характеристики и экспрессию менина в 169 спорадических НЭО ПЖЖ. Исследователи выявили ядерную экспрессию и слабую цитоплазматическую экспрессию менина в нормальных тканях ПЖЖ, тогда как в 80% НЭО экспрессия была аномальной (135/140 — цитоплазматическая; 111/140 — ядерная; 5/140 — отсутствие экспрессии). В 27 НЭО ПЖЖ выявлена мутация в MEN1(25 соматических и 2 герминальных мутации); в 5 НЭО ПЖЖ отсутствовала экспрессия менина и в ядре, и в цитоплазме. Авторы отметили, что в половине случаев с отсутствием ядерной экспрессии менина и в трети случаев со слабой ядерной экспрессией и умеренной или интенсивной цитоплазматической экспрессией имелись мутации в MEN1. Они предположили, что некоторые из мутаций вызывают преждевременную блокировку транскрипции, образовывая патологический белок, который накапливается в цитоплазме из-за отсутствия по крайней мере одного из сигналов ядерной локализации менина. Мутации, не приводящие к образованию патологического белка, также могут частично влиять на ядерный транспорт, возможно, вызывая неполный процессинг белка или препятствуя взаимодействию менина с другими белками, что может объяснить обилие цитоплазматической экспрессии менина, наблюдаемого при наличии мутации в MEN1 [21]. Гипотеза была подтверждена вестерн-блоттингом, демонстрирующим экспрессию менина в опухолях с мутациями в гене MEN1.

Y. Cao и соавт. продемонстрировали in vitro, что менин способен перемещаться между ядром и цитоплазмой посредством ядерных экспортных сигналов. Что еще более интересно, менин напрямую взаимодействует с β-катенином (Wnt/β-катенин сигнальный путь) и при сверхэкспрессии менина предотвращает накопление в ядре β-катенина, выводя белок за пределы ядра. Они продемонстрировали, что мутации в доменах, ответственных за ядерные экспортные сигналы, могут нарушать экспортную функцию менина. Эти и результаты других исследований предполагают, что патологические состояния, затрагивающие функции менина, могут изменять клеточную локализацию его интерактора (например, β-катенина) и позволяют сделать предположение о том, что аномальная экспрессия менина может возникнуть в случае каких-либо изменений со стороны его интерактора (например, опухоли могут накапливать другие мутации, которые способны изменять экспрессию интерактора) [22–24].

В регуляции экспрессии менина принимают участие и другие сигнальные пути. Соматостатин усиливает экспрессию менина посредством подавления рецептора SSTR2A цАМФ-PKA сигнального пути [24]. Кратковременная стимуляция глюкозой подавляет экспрессию менина посредством фосфорилирования и ингибирования фактора транскрипции FOXO1 через сигнальный путь PI3K/Akt [25], а K-Ras подавляет экспрессию менина, способствуя связыванию DNMT1 с промотором гена MEN1 и увеличивая метилирование ДНК, тогда как менин ингибирует Ras-опосредованную передачу сигналов [26]. Эпигенетическая регуляция экспрессии менина также может происходить посредством микро-РНК, например, при связывании miR-24-1 с 3’-нетранслируемой областью (3’-UTR) мРНК менина подавляется его экспрессия [27][28]. Кроме того, имеются посттрансляционные модификации менина, включая сумоилирование по лизину 591, фосфорилирование по серину 394 и быструю деградацию с помощью убиквитин-протеосомного пути [11].

Как упоминалось ранее, мутации в других ассоциированных генах способствуют изменению экспрессии менина. K. Lines и соавт. в своем исследовании оценили потенциальную роль генетических модификаторов в развитии опухолей у взрослых мышей Men1+/-, так как определение таких модификаторов может обеспечить лучшее понимание функции менина и его молекулярных взаимодействий в эндокринных опухолях, а также предоставить инструмент для прогнозирования появления опухолей у пациентов с мутациями в гене MEN1 и новые мишени как для моно-, так и для комбинированной терапии. В общей сложности были изучены 275 мышей Men1+/- в возрасте 5–26 мес конгенных штаммов C57BL/6 и 129S6/SvEv. Исследователи выявили, что опухоли гипофиза и надпочечников развивались раньше у самцов C57BL/6 и самок 129S6/SvEv соответственно, а опухоли поджелудочной железы и яичек развивались раньше у самцов 129S6/SvEv. По данным ИГХ-исследования во всех опухолях отсутствовала экспрессия менина. При анализе последовательности всего генома мышей 129S6/SvEv и C57BL 6 Men1+/- выявили >54 000 различных вариантов модификаторов в >300 генах. Выявленные гены (Kras, Wnt2b, Il3ra и Tnfrsf10a) связаны с сигнальными путями, участвующими в опухолеобразовании, и потенциально могут быть модификаторами MEN1, поскольку менин подавляет MAPK-управляемую пролиферацию KRAS, контролирует передачу сигналов wnt посредством взаимодействия с β-катенином, регулирует экспрессию интерлейкинов и способствует ФНО-α апоптозу за счет активации каспазы 8 [29].

Также обращает на себя внимание, что аутопсийный гипофиз и нормальная ткань гипофиза в поле зрения АГ, полученные после операции, имели только цитоплазматическую экспрессию. Самая слабая экспрессия была обнаружена в нормальных гипофизах. По данным исследований, это может быть результатом посмертной деградации белка в ядре [16].

Наша работа имела некоторые ограничения: исследование было ретроспективным с малым количеством образцов в выборке ввиду редко встречающихся патологий. Также существует небольшая вероятность того, что пациенты с мутациями в гене MEN1 могли быть включены в спорадическую группу. Для полного понимания картины необходимы дальнейшие исследования с определением соматических мутаций и потерей гетерозиготности гена MEN1, а также исследование эпигенетических факторов, сигнальных путей, в которых участвует менин.

## ЗАКЛЮЧЕНИЕ

По результатам исследования оценки экспрессии менина в образцах тканей АГ в группах с гМЭН 1, фМЭН 1 и СА было выявлено, что экспрессия менина в целом сохранена в АГ у фМЭН 1 и спорадических соматотропиномах, хотя и с разной локализацией в структуре клетки (ядро и/или цитоплазма). В то же время экспрессия менина сильно варьирует в АГ у пациентов с истинным синдромом МЭН 1. По полученным данным можно предположить, что патогенез АГ при фМЭН 1 и СА может иметь сходства, однако при фМЭН 1, по всей видимости, имеются факторы, которые способствуют возникновению нескольких опухолей эндокринных желез у одного человека. Для понимания процесса необходимо дальнейшее исследование ассоциированных с МЭН 1 генов, эпигенетических факторов, сигнальных путей, в которых участвует менин.

## ДОПОЛНИТЕЛЬНАЯ ИНФОРМАЦИЯ

Источник финансирования. Исследование выполнено в рамках гранта Президента Российской Федерации для государственной поддержки молодых российских ученых — кандидатов наук № МК-1100.2020.7 «Морфологические и молекулярно-генетические особенности аденом гипофиза в рамках фенокопий синдрома множественных эндокринных неоплазий 1 типа».

Конфликт интересов. Авторы декларируют отсутствие явных и потенциальных конфликтов интересов, связанных с публикацией настоящей статьи.

Участие авторов. Все авторы внесли значимый вклад в проведение исследования и подготовку статьи, прочли и одобрили финальную версию статьи перед публикацией.
